# Effects of Cation Exchange in Rhodamine B Photocatalytic Degradation Using Peroxo-Titanate Nanotubes

**DOI:** 10.3390/nano14141170

**Published:** 2024-07-09

**Authors:** Do Hyung Han, Hyunsu Park, Tomoyo Goto, Sunghun Cho, Yeongjun Seo, Yoshifumi Kondo, Hisataka Nishida, Tohru Sekino

**Affiliations:** 1 Department of Advanced Hard Materials, SANKEN, Osaka University, 8-1 Mihogaoka, Ibaraki 567-0047, Japan; dh.han23@sanken.osaka-u.ac.jp (D.H.H.); hspark23@sanken.osaka-u.ac.jp (H.P.); goto@sanken.osaka-u.ac.jp (T.G.); shcho@sanken.osaka-u.ac.jp (S.C.); yjseo@sanken.osaka-u.ac.jp (Y.S.); y.kondo@sanken.osaka-u.ac.jp (Y.K.); hnishida@sanken.osaka-u.ac.jp (H.N.); 2 Institute for Advanced Co-Creation Studies, Osaka University, 1-1 Yamadaoka, Suita 565-0871, Japan

**Keywords:** layered sodium titanate, peroxo-titanate nanotubes, Rhodamine B dye, cation exchange, photocatalytic degradation

## Abstract

Lepidocrocite-type layered sodium titanate (Na_x_H_2−x_Ti_2_O_5_) is widely used in environmental remediation because of its large specific surface area, formed by anisotropic crystal growth, and its ability to store and exchange cations between layers. Additionally, peroxo-titanate nanotubes (PTNTs), which are tubular titanates with peroxy groups, exhibit visible-light absorption capabilities, rendering them suitable for photocatalytic applications under visible light irradiation. However, because of cation exchange reactions, the Na^+^ concentration and pH of the solution can fluctuate under aqueous conditions, affecting the photocatalytic performance of the PTNTs. Herein, we evaluated the impact of cation exchange reactions on the photocatalytic degradation of Rhodamine B (Rh B) by PTNTs at controlled Na^+^ ratios. The observed pH of Rh B solutions increases due to the cation exchange reaction with Na^+^ and H_3_O^+^, leading to the formation of zwitter-ionic Rh B molecules, eventually weakening their adsorption and photodegradation performance. Moreover, the results indicate that inhibiting the pH increase of the Rh B solution can prevent the weakening of both the adsorption and photodegradation performance of PTNTs. This study highlights the significance of regulating the sodium ion content in layered titanate materials, emphasizing their importance in optimizing these materials’ photocatalytic efficacy for environmental purification applications.

## 1. Introduction

Among semiconductor materials, titanium oxide stands out for its high chemical stability and non-toxicity, making it a widely studied substance for the environmental purification of water and air pollution [1,2,3,4]. In particular, research on the photocatalytic degradation of pollutants such as dyes and pigments, which contribute to water contamination, has been ongoing for decades [5,6,7]. Among the series of titanium-based compounds, alkaline titanate materials have also been studied because of their varied crystal structures and excellent properties, such as photocatalytic reactions and cation exchange [8,9,10]. In particular, lepidocrocite-type sodium titanate (Na_2_Ti_n_O_2n+1_) shows a layered structure, in which TiO_6_ octahedra are shared at the edges and corners and have Na^+^ between interlayers [11,12,13]. By removing the Na^+^ between the layers of this material, titanate nanotubes, which possess a large specific surface area derived from a unique nanotubular structure formed by the scrolling of an anisotropic crystal-grown layer structure, can be obtained [14,15,16,17]. Focusing on the photocatalytic properties, the large specific surface area of the material can enhance photocatalytic performance by increasing the contact area with the pollutants [18,19,20]. Furthermore, the cations present in the interlayer of the layered crystal structure are exchangeable, making them suitable for the environmental purification of radioactive substances or heavy metal pollutants [21,22]. However, the bandgap energy of titanate nanotubes is 3.5 eV, limiting their photocatalytic application to conditions where ultraviolet light exceeds the bandgap energy, thus hindering their ability to harness visible light [23,24].

To solve this problem, our research group reported the synthesis of layered peroxo-titanate nanotubes (PTNTs) using ion precursors and a bottom-up process [25,26]. In previously reported synthesis methods, peroxo titanates were often synthesized by adding H_2_O_2_ after reacting with raw titanium materials [27,28]. Park et al. [25] introduced a method for the synthesis of peroxo titanates via the self-assembly of peroxo-titanium complex ions (PTC ion, Ti[(OH)_3_O_2_]^−^). This method enables their utilization as visible light-responsive photocatalytic materials owing to the inclusion of peroxo bonding within their structure, yielding a bandgap energy of approximately 2.5 eV [25,26]. However, titanate materials with layered crystal structures undergo various cation exchange reactions in aqueous solutions, leading to unpredictable interactions with different ions. In particular, as the cation exchange reaction of the Na^+^ remaining between the layers progresses in the solution, it can result in changes in the solution’s pH, depending on the concentration of H_3_O^+^. Moreover, changes in the solution pH can alter the characteristics of a material, affecting its photocatalytic performance [29]. For instance, the surface charge of TiO_2_ in solution exhibits an isoelectric point at pH 6–7, with a positive charge under acidic conditions and a negative charge under alkaline conditions [30]. These changes can affect the adsorption characteristics of TiO_2_ for other materials (such as pollutants, in the case of photocatalysts), thereby affecting the photocatalytic performance. Therefore, it is important to understand the phenomena occurring in solution due to the ion-exchange properties of PTNT materials and simultaneously study their effects on the photocatalytic performance.

In this study, PTNT materials with different compositions of Na^+^ in the interlayer structure were prepared by adjusting the synthesis conditions, and their crystal structure, chemical composition, morphology, and optical properties were evaluated to understand their relationship with their photocatalytic properties. Furthermore, the photocatalytic performances of PTNT materials with different ion compositions were evaluated using Rhodamine B (Rh B) organic dye as a model material for organic pollutants to assess the effects of ion-exchange reactions on changes in the Rh B solution and their influence on photocatalytic performance.

## 2. Materials and Methods

### 2.1. Synthesis of Peroxo-Titanium Complex (PTC) Ion Precursors

The PTC ion precursors were synthesized following the procedures outlined in a previous study [19]. A solution comprising 62.5 mL of H_2_O_2_ (30%, FUJIFILM Wako Pure Chemical Laboratory Corporation, Osaka, Japan) and 15.29 mL of 10 mol/L NaOH (97%, FUJIFILM Wako Pure Chemical Laboratory Corporation, Osaka, Japan) was prepared to achieve a NaOH concentration of 1.5 mol/L and pH = 10. Subsequently, 1.87 g of TiH_2_ powder (>99%, Kojundo Chemical Laboratory Co., Ltd., Saitama, Japan) was added to the prepared mixture (NaOH and H_2_O_2_) and allowed to ionize for 24 h.

### 2.2. Synthesis of Layered Peroxo-Titanate Nanotube (PTNT)

**Figure 1:**
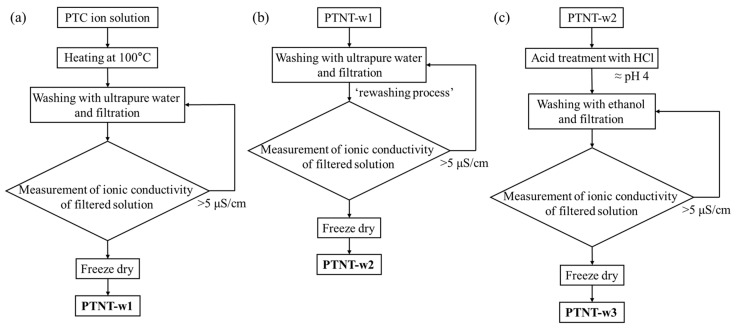
Schematics diagram of preparation process for (**a**) PTNT-w1, (**b**) PTNT-w2, and (**c**) PTNT-w3.

Figure 1 shows a schematic of the preparation process of the PTNTs samples in this study. The PTC ion solution was subjected to heating at 100 °C for 24 h with stirring at 200 rpm in a refluxing vessel at atmospheric pressure. Upon completion of the reaction, the resulting precipitates were subjected to multiple rounds of washing with ultrapure water and filtration using a vacuum pump (MDA-020C, ULVAC, Inc., Kanagawa, Japan) until the ionic conductivity of the filtered solution reached approximately 5 μS/cm. Subsequently, the washed samples were dried using a freeze dryer (EYELA FDU-2200, Tokyo Rikakikai Co., Ltd., Tokyo, Japan) and labeled as ‘PTNT-w1’ (Figure 1a). Furthermore, to further remove the Na^+^ ions present in the PTNT-w1 sample, the dried PTNT-w1 sample was immersed in ultrapure water. The PTNT-w1 sample underwent a ‘rewashing process’, additional rounds of washing with ultrapure water and filtration using a vacuum pump, until the ionic conductivity of the filtered solution reached approximately 5 μS/cm. Following this rewashing process, the collected samples were dried using a freeze dryer using the same method as described above and labeled as ‘PTNT-w2’ (Figure 1b). To remove more Na^+^ ions from the PTNT-w2 sample, the dried PTNT-w2 sample was mixed with ultrapure water, followed by the addition of 0.01 mol/L HCl (hydrochloric acid, 35–37%, FUJIFILM Wako Pure Chemical Laboratory Corporation, Osaka, Japan) solution until the solution reached pH 4. The mixture was then stirred at 200 rpm for 2 h. Subsequently, the mixture was repeatedly washed with ethanol (99.5%, FUJIFILM Wako Pure Chemical Laboratory Corporation, Osaka, Japan), filtered using a vacuum pump, and the washing process was continued until the ionic conductivity of the filtered solution reached 5 μS/cm. The collected sample after additional acid treatment was dried using a freeze dryer by the same method above and labeled as ‘PTNT-w3’ (Figure 1c).

### 2.3. Characterization of PTNT Samples

The crystal phases of the samples were identified by X-ray diffraction (XRD; D8 ADVANCE, Bruker AXS Co. Ltd., Karlsruhe, Germany). XRD patterns were obtained using a Scintag diffractometer, operated in the Bragg configuration with Cu Kα radiation (λ = 1.54 Å) from 5.0 to 85.0°, at a scanning rate of 0.02°. X-ray fluorescence (XRF) spectroscopy (ZSX100e, Rigaku Co., Tokyo, Japan) was employed to determine the molar ratios of Na and Ti in the titanate samples in the EZ scan mode. For the morphological analysis, field-emission scanning electron microscopy (FE–SEM, SU-9000, Hitachi High-Tech Corp., Tokyo, Japan) and scanning transmission electron microscopy (STEM) were performed at an acceleration voltage of 30 kV. The specific surface area was calculated using the Brunauer–Emmett–Teller (BET) method based on the adsorption isotherm obtained in the P/P_0_ range of 0.1–0.3, measured using an N_2_ adsorption–desorption instrument (NOVA 4200e, Quantachrome Instruments, Boynton Beach, FL, USA). Optical properties such as reflectance and bandgap energies were analyzed using ultraviolet–visible (UV–vis.) spectroscopy (V-650, JASCO Corp., Tokyo, Japan). The zeta potential was evaluated using a dynamic light scattering analyzer coupled using a laser Doppler microelectrophoresis method (Zetasizer, Nano-ZS MALVERN Panalytical, Worcestershire, UK), with each measurement repeated three times to ensure accuracy. The pH of the solution was adjusted with a buffer solution and monitored using a pH meter (D-52; HORIBA Ltd., Kyoto, Japan).

### 2.4. Photocatalytic Degradation Study of PTNT Samples

The photocatalytic properties of the PTNT samples were assessed through a photodegradation reaction using Rh B (Sigma-Aldrich Japan, Tokyo, Japan) and Tetracycline (TC, FUJIFILM Wako Pure Chemical Laboratory Corporation, Osaka, Japan) as organic compounds. Rh B and TC solutions with concentrations of 10 mg/L were prepared, and each test was conducted in an aqueous phase in a stirred batch reactor containing 2 g/L of the photocatalyst, suspended in 50 mL of the substrate solution. The solution was stirred at 300 rpm in the dark for 30 min to achieve the adsorption–desorption equilibrium of the organic molecules on the PTNT surface. The solution was then stirred and irradiated with controlled UV and visible light. UV light was applied using a UV irradiator (TOSCURE 100, Toshiba Light-Tech. Co. Ltd., Yokosuka, Japan). The wavelength was limited to 280–380 nm by using a U340 bandpass filter (Hoya Corporation, Tokyo, Japan). By contrast, visible light from a solar simulator (OTENTO-SUN III, Bunkoh-Keiki Co. Ltd., Tokyo, Japan) was calibrated at a standard air mass of 1.5 and 1000 W/m^2^. Wavelengths below 420 nm were eliminated using an L-42 bandpass filter (<420 ± 5 nm, Hoya Corporation, Tokyo, Japan). The reacted solution was sampled at time intervals of 60 min and filtered using a 0.2 μm pore-sized filter to remove the powder, and their UV−vis. absorption spectra were recorded. The concentrations of Rh B and TC were determined from their characteristic absorbance peaks at 553 and 357 nm, respectively. The degradation rate (%) of organic compounds was calculated using Equation (1).

(1) $$\mathrm{Degradation}\;\%=\frac{C_{0}-C_{t}}{C_{0}}\times 100,$$

where *C*_0_ and *C_t_* are the absorbances of the Rh B and TC solutions before and after the photocatalytic reaction, respectively.

To know the influence of the solution pH under the test, the concentration of Rh B and TC solutions and the amount of the added PTNT-w2 sample were kept consistent. After mixing the PTNT-w2 sample, 0.01 mol/L HCl solution was added to adjust the pH to 4, and the solution was stirred at 300 rpm in darkness for 30 min to achieve adsorption–desorption equilibrium. The solution was stirred and irradiated with controlled UV or visible light.

### 2.5. Kinetic Study of Rh B Photodegradation Test

The photodegradation kinetics of Rh B were studied using the Langmuir–Hinshelwood model [31,32]. The rate constants (*k*) were calculated using the Rh B concentration after 30 min of adsorption–desorption equilibrium under dark conditions.

(2) $$\ln C_{t}=\ln C_{0}-kt$$

(3) $$\ln\left(\frac{C_{t}}{C_{0}}\right)=-kt$$

where *C_t_* is the Rh B concentration in the sample collected after time *t*, *C*_0_ is the initial Rh B concentration, and *k* is the photodegradation rate constant.

## 3. Results and Discussion

### 3.1. Crystallographic Properties of PTNT Samples

**Figure 2:**
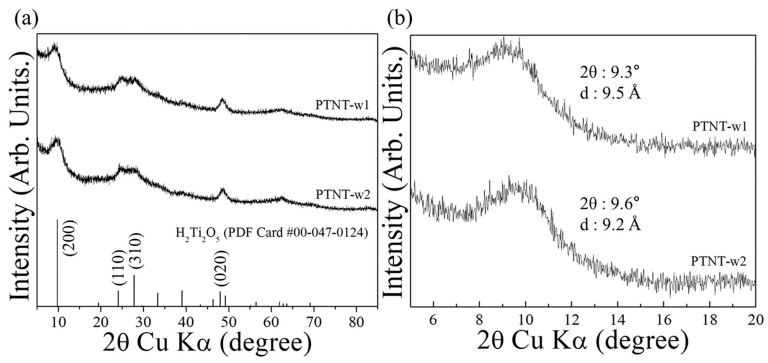
(**a**) XRD pattern of PTNT-w1 and PTNT-w2 samples and (**b**) enlarged graph of the 200 diffraction peak in (**a**).

Figure 2a shows the XRD pattern of the PTNT-w1 and PTNT-w2 samples. The XRD patterns showed that all PTNT samples were identified as the layered titanate phase (H_2_Ti_2_O_5_) (PDF Card #00-047-0124). In the XRD pattern of the titanate phase, the 2*θ* and *d*-spacing values of the 200 diffraction peak are associated with the interlayer distance, which is determined by the type and amount of cations (such as Li^+^, Na^+^, and K^+^) between the layers. The *d*-spacing value of the 200 diffraction peak (*d*_200_) can be calculated using Bragg’s law (Equations (4) and (5)):

(4) nλ=2dsin⁡θ

(5) $$d_{200}=\frac{\lambda }{2\sin\theta }$$

where *n* is the order of reflection, *λ* is the wavelength of X-ray radiation, *d* is the inter-planar spacing distance, and *θ* is the diffraction angle. Figure 2b shows an enlarged view of the 200 diffraction peak region, and Table 1 summarizes the 2*θ* and *d*-spacing values obtained by XRD results and the chemical composition of the each PTNT sample calculated based on the results of the XRF analysis. As shown in Figure 2b and Table 1, the PTNT-w2 sample, subjected to additional washing steps compared to PTNT-w1, exhibited a smaller interlayer distance. This phenomenon indicates that the exchange of Na^+^ ions with H^+^ (H_3_O^+^) ions occurs upon washing with ultrapure water. Moreover, from the XRF results, the chemical composition was calculated to have changed from Na_0.5_H_1.5_Ti_2_O_5_ for PTNT-w1 to Na_0.2_H_1.8_Ti_2_O_5_ after the ‘rewashing process’. That is, within the interlayer spaces of PTNT, the number of larger Na^+^ ions probably decreased, as they were exchanged with smaller H^+^, ions based on their hydrated ionic radius.

### 3.2. Morphological Properties of the PTNT Samples

**Figure 3:**
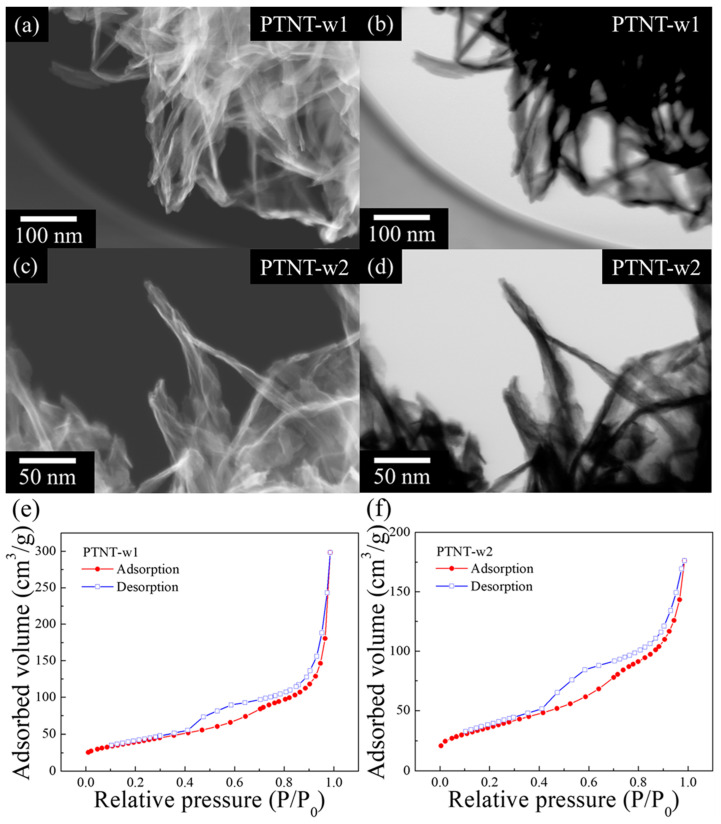
(**a**–**d**) SEM and STEM images and (**e**,**f**) N_2_ adsorption–desorption isotherm graphs of PTNT-w1 (**a**,**b**,**e**) and PTNT-w2 (**c**,**d**,**f**) samples.

Figure 3 shows SEM and STEM images and the N_2_ adsorption–desorption isotherms of PTNT-w1 (a, b, and e) and PTNT-w2 (c, d, and f). From the SEM and STEM images (Figure 3a–d), both samples demonstrated tubule-like structures formed by agglomerated or scrolled sheets, consistent with previously reported findings on PTNT [25]. The isotherms in Figure 3e,f show that both PTNT samples exhibit type IV behavior with H3-type hysteresis loops, according to the International Union of Pure and Applied Chemistry (IUPAC) classification. Table 2 presents the specific surface area of each sample, calculated using the Brunauer–Emmett–Teller (BET) method based on the N_2_ adsorption–desorption isotherm. These results revealed no significant observable difference in the morphological characteristics between PTNT-w1 and PTNT-w2. Next, Rh B photocatalytic degradation tests were conducted using two PTNT materials with different amounts of Na^+^.

### 3.3. Photocatalytic Degradation of Rhodamine B

#### 3.3.1. Effects of Sodium Ion Contents on Photocatalytic Properties

**Figure 4:**
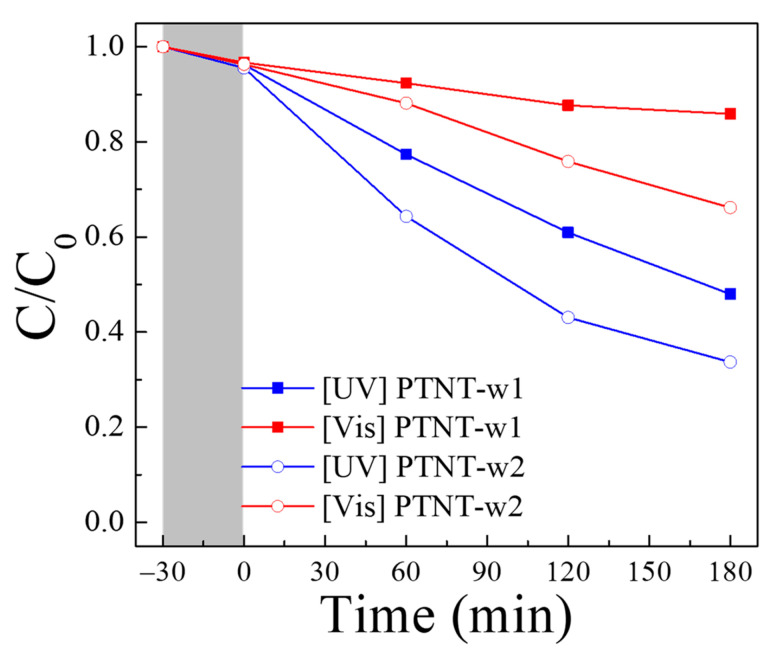
Photocatalytic degradation results of Rh B using PTNT-w1 and PTNT-w2 samples UV and vis. light irradiation. The gray area corresponds to dark condition prior to light irradiation.

Figure 4 shows the results of the photocatalytic degradation test of Rh B using the PTNT-w1 and PTNT-w2 samples. Both samples exhibited an adsorption of approximately 4–5% of Rh B during the initial 30 min under dark conditions. Subsequently, under UV light, the 3 h photodegradation reactions using PTNT-w1 and PTNT-w2 resulted in the decomposition of 52% and 66% of Rh B, respectively, while under visible (vis.) light, 14% and 33% of Rh B were decomposed, respectively. PTNT-w2 exhibited more pronounced degradation regardless of the UV or vis. light source. Furthermore, photodegradation occurred faster under UV irradiation than under visible light, regardless of the number of Na^+^ ions present. As mentioned in the introduction, the photocatalytic performance of a catalyst is significantly influenced by its specific surface area and photocatalytic reactivity. However, as shown in Figure 3, both samples show approximately the same surface area, and therefore, PTNT-w1 and PTNT-w2, with different amounts of Na^+^ ions, had their optical characteristics evaluated.

**Figure 5:**
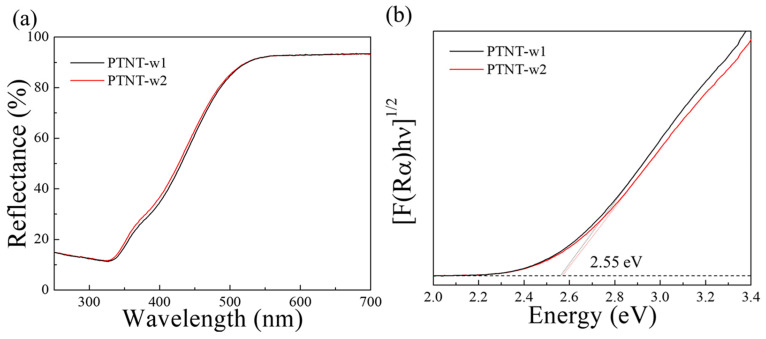
(**a**) UV–vis. reflectance spectra and (**b**) Tauc plots of PTNT-w1 and PTNT-w2 samples.

Figure 5 shows the UV–vis. reflectance spectra and Tauc plots of PTNT-w1 and PTNT-w2 samples calculated by considering indirectly allowed transition. Both samples absorbed light below 550 nm, with a band gap energy, calculated based on the Kubelka–Munk theory, of 2.55 eV [33,34]. Similar to their morphological characteristics, there is no significant difference in UV–vis. reflectance spectra between PTNT-w1 and PTNT-w2, consistent with previous reports on the characteristics of PTNT materials [25]. These results suggest that even with variations in the ratio of Na^+^ ion contents from 0.5 to 0.2 in PTNTs, although differences arise in the photodegradation performance of Rh B, there is no significant difference in the morphological and optical properties of the PTNT.

#### 3.3.2. Effect of Cation Exchange on Rh B Solution

In general, layered titanate possesses cation exchange ability, which can affect the solution pH, potentially impacting the photodegradation performance. Thus, the pH values of the Rh B solutions containing the PTNT-w1 and PTNT-w2 samples were measured. The Rh B solution (10 mg/L) had an acidic pH value of 4.0. However, when the PTNT-w1 or PTNT-w2 samples were added, each of the resulting solutions had pH values of 7.9 and 7.1, respectively, showing a slight increase. These changes were probably due to cation exchange occurring when the PTNT containing Na^+^ ions and an acidic Rh B solution were mixed. The exchange of H^+^ ions from the acidic Rh B solution with Na^+^ ions from PTNT increased the solution’s pH. Moreover, a higher Na content in PTNT material correlates with a greater increase in pH. Therefore, in the case of PTNT-w2, which showed a smaller pH increase, the effect of ion exchange on the photodegradation performance was assumed to be small. To investigate this hypothesis, the PTNT-w3 sample, which had the lowest Na^+^ content, was prepared, and its photodegradation performance was evaluated.

### 3.4. Methods for Minimizing the Influence of Cation Exchange

#### 3.4.1. Minimizing Sodium Ion Contents in PTNT Samples

**Figure 6:**
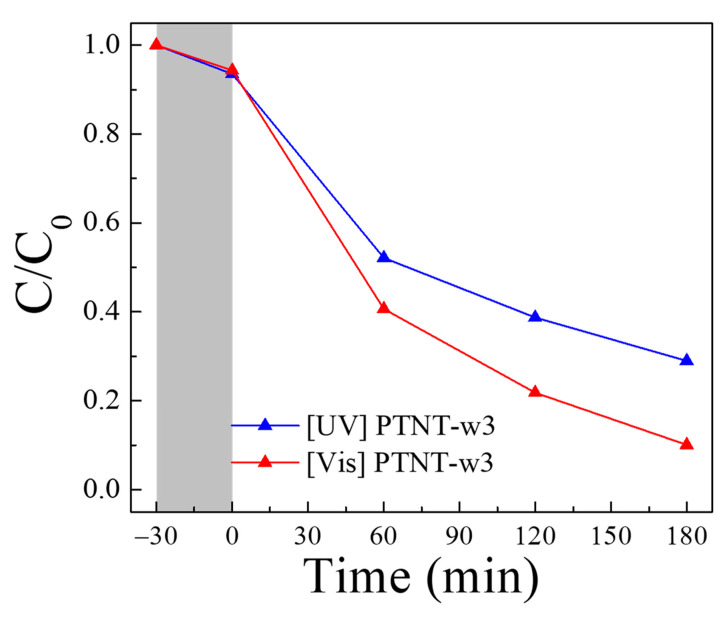
Photocatalytic degradation results of Rh B using PTNT-w3 sample under the UV and vis. light irradiation. The gray area corresponds to dark condition prior to light irradiation.

Figure 6 shows the Rh B photodegradation results using the PTNT-w3 sample. Under dark conditions, the adsorption capacity after 30 min was 6–7%. The Rh B degradation reached 71% and 90% over 3 h under UV and vis. light sources, respectively. Table 3 presents the chemical composition and specific surface area of the PTNT-w3 sample. The chemical composition of PTNT-w3 is Na_0.1_H_1.9_Ti_2_O_5_, indicating a Na^+^ ion content approximately half of that in PTNT-w2. Upon mixing with the Rh B solution, the pH was 5.0. Comparing the Rh B adsorption of PTNT-w3 with those of PTNT-w1 and PTNT-w2, there was no significant difference in specific surface area; however, the adsorption capacity for 30 min under dark conditions was approximately doubled. Furthermore, the Rh B photodegradation reaction over 3 h under both UV and vis. light sources proceeded more effectively with PTNT-w3. Therefore, to examine the influence of the solution’s pH on the adsorption and photodegradation reactions in more detail, conditions with reduced pH were also tested. In this approach, the solution’s pH was adjusted using an HCl solution rather than adjusting the Na^+^ ion content of PTNTs, and this sample was designated as ‘PTNT-w2 + HCl’.

#### 3.4.2. Controlling the pH of the Rhodamine B Solution

**Figure 7:**
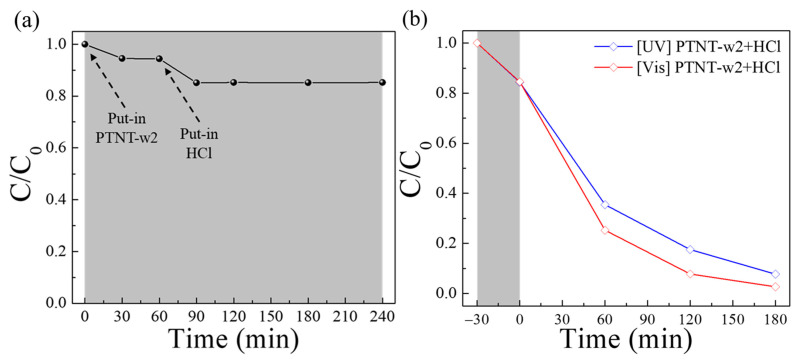
(**a**) Changes in Rh B concentration (*C/C*_0_) under the dark conditions when PTNT-w2 was inserted, before and after the addition of HCl solution, and (**b**) photocatalytic degradation results of Rh B under the PTNT-w2 + HCl condition, where the gray area corresponds to dark condition before UV and vis. light irradiation.

Figure 7a shows the changes in Rh B concentration (*C/C*_0_) when the PTNT-w2 and HCl solutions were added to the Rh B solution under dark conditions. At the beginning of the reaction, as indicated by the rate of decrease in the Rh B concentration upon adding PTNT-w2, an approximate 5% reduction was observed. This reduction tripled to about 15% after adding the HCl solution. Subsequently, the Rh B concentration remained constant for 3 h, with no significant differences, due to the addition of HCl. Figure 7b shows the Rh B photodegradation result using the PTNT-w2 + HCl condition. Similar to Figure 7a, an approximately 15% decrease in the Rh B concentration was observed in the dark for 30 min. Under UV and visible light irradiation for 3 h, the Rh B degradation reached approximately 92% and 97%, respectively. Comparing these results with those obtained under different conditions (PTNT-w1, PTNT-w2, and PTNT-w3), the most superior adsorption and photodegradation performance were observed under the PTNT-w2 + HCl condition. Of particular interest are two key points: first, the enhancement in the adsorption reaction of Rh B and second, the increase in the photocatalytic degradation rate of PTNT.

**Figure 8:**
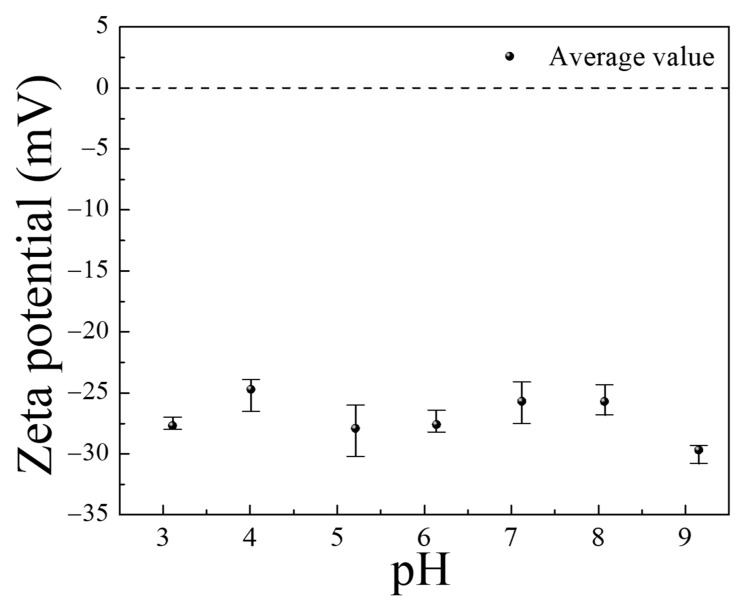
Changes in zeta potential against the solution pH of the PTNT-w2 sample.

Regarding the enhancement in adsorption capability, it is necessary to investigate the phenomena arising from the changes in the PTNT surface properties, as well as those occurring owing to variations in the Rh B solution characteristics. Figure 8 shows the results of the zeta potential measurements for the PTNT material (PTNT-w2), which are relevant to the adsorption capacity concerning pH variations. In general, adsorption performance is influenced by the surface charge of the photocatalyst, as explained by the zeta potential [22]. For PTNT-w2, it was found that there was no significant change in the zeta potential, ranging from −30 to −25, across the pH range (pH 3–8) tested in this study. Therefore, it was concluded that the variation in the adsorption properties between the Rh B molecules and PTNT was likely due to factors other than changes in the surface properties of the PTNT material.

**Figure 9:**
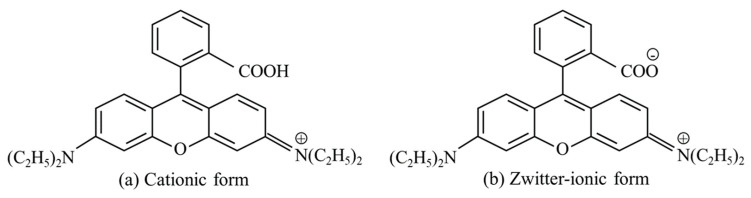
Ionic forms of Rh B: (**a**) cationic form and (**b**) zwitter-ionic form.

Therefore, the variation in the ionic form of Rh B in the solution with respect to pH was considered. Figure 9 shows the two ionic forms of the Rh B molecule: (a) cationic form and (b) zwitter-ionic form. The Rh B solution inherently possesses an acidic form, wherein the ionic form of the Rh B molecules resembles that shown in Figure 9a, with abundant H^+^ ions attached to the carboxyl group (–COOH), and the nitrogen of the amino group carrying a positive charge, resulting in an overall positive charge on the Rh B molecule [35]. However, as the pH increases, the Rh B molecules transform into their zwitter-ionic forms, as shown in Figure 9b. In the zwitter-ionic form, the carboxyl group (–COO^−^) is negatively charged, whereas the amino group is positively charged. Inyinbor et al. and Zamouche et al. reported that Rh B molecules in zwitter-ionic form tend to aggregate, hindering adsorption [35,36]. Therefore, in this study, it was anticipated that the Rh B molecules would be transformed into the zwitter-ionic form by increasing the pH, owing to the addition of the PTNT sample into the Rh B solution. In the case of PTNT-w1 and PTNT-w2, the limited adsorption of Rh B resulted in a poor photocatalytic reaction. However, by adjusting the amount of Na^+^ ions present in PTNT, as in the case of the PTNT-w3, or by controlling pH, as in the PTNT-w2 + HCl condition, the formation of the zwitter-ionic form of Rh B could be reduced, thereby enhancing adsorption and photodegradation performance.

### 3.5. Kinetic Study of Rh B Photodegradation

**Figure 10:**
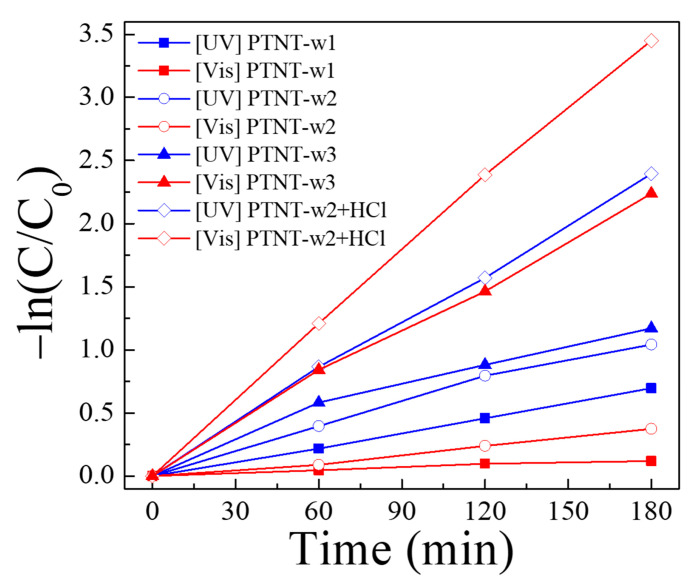
Kinetics graph of the photodegradation using all PTNT samples.

In addition, the kinetics of all the photocatalytic performances evaluated in this study were analyzed according to the Langmuir–Hinshelwood model described in Equation (3). Figure 10 and Table 4 present the kinetics and calculated rate constant (*k*) values for all photocatalytic reactions under UV and visible light for the PTNT-w1, PTNT-w2, PTNT-w3, and PTNT-w2 + HCl conditions. The rate constants were calculated based on the Rh B concentration after photocatalytic degradation over time, following 30 min in the dark, and showed a linear relationship (R-squared value > 0.95). In the case of PTNT-w2 + HCl, the rate constants for UV and vis. light sources were 2.19 × 10^−4^ s^−1^ and 3.20 × 10^−4^ s^−1^, respectively, showing that the fastest degradation of Rh B is independent of the type of light source. Particularly noteworthy is that for PTNT-w1 and PTNT-w2, the rate constants under UV were faster than those under the vis. light source. Conversely, for PTNT-w3 and PTNT-w2 + HCl, the rate constants under vis. light were higher than those under the UV light source. Consequently, although the adsorption of Rh B on PTNT was poor (in PTNT-w1 and PTNT-w2), the photocatalytic reaction was faster under UV compared to vis. light. However, in cases where adsorption was enhanced (in PTNT-w3 and PTNT-w2 + HCl), the photocatalytic reaction was faster under vis. light compared to UV. According to Chen et al. [30], under vis. light, active oxygen species are generated around the adsorption sites of the photocatalyst material, facilitating photocatalytic degradation. Conversely, under UV light, the availability of higher energy with short wavelengths leads to the generation of active oxygen species, regardless of the adsorption sites on the photocatalyst [30]. From these results, it can be inferred that, depending on the type of light source, the relationship between the photocatalytic reaction rates of PTNTs, the adsorption state, and the amount of Rh B is reversed.

### 3.6. Effects of Cation Exchange in TC Photodegradation Test

**Figure 11:**
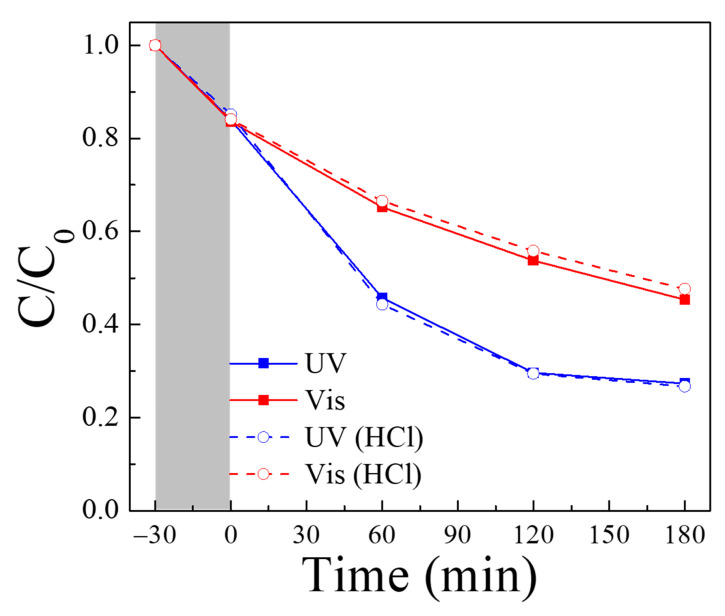
Photocatalytic degradation of TC using PTNT-w2 sample under the UV and vis. light irradiation, where the test was carried out under the normal (square) and pH adjusted using HCl (circle) conditions. The gray area corresponds to the dark condition without light irradiation.

Finally, to assess whether factors other than the pH-induced ionic form-change of Rh B affected the photodegradation performance observed in this study, TC organic pollutants were evaluated instead of Rh B in the photodegradation test. Initially, the TC solution had a pH of 3.5, similar to that of the Rh B solution. However, similar to the Rh B solution, due to cation exchange reactions induced by the PTNT-w2 addition, the pH increased to 5.6. Figure 11 shows the photocatalytic degradation results of the TC solution after adding the PTNT-w2 sample, leading to an increase in pH, and after the adjustment of pH by adding HCl. There were no significant differences in the adsorption and photodegradation reaction rates with or without HCl addition after 30 min in the dark. Consequently, the decrease in the adsorption amount and photodegradation rate due to the increase in pH caused by the Rh B solution and PTNT addition is primarily attributed to the variation in the ionic form of Rh B molecules with pH.

## 4. Conclusions

In this study, the adsorption and photodegradation performance of PTNT samples was evaluated using Rh B and TC solutions. In particular, focusing on the influence of the cation exchange ability of the PTNT material, Rh B photodegradation tests were conducted while observing the pH changes in the Rh B solution based on the amount of Na^+^ ions present in PTNTs. Even with variations in the Na^+^ ion content of the PTNT materials ranging from Na_0.5_H_1.5_Ti_2_O_5_ to Na_0.1_H_1.9_Ti_2_O_5_, there were no significant differences in their morphological and optical properties. However, the adsorption and photodegradation rates of Rh B varied for each sample, primarily because of the change in the ionic form of Rh B with pH. When the PTNT materials containing Na^+^ ions were mixed with an acidic Rh B solution, a cation exchange reaction occurred, causing an increase in the pH of the Rh B solution. Consequently, it was estimated that the Rh B molecules transformed from a cationic form to a zwitter-ionic form, leading to poor adsorption due to the aggregation of the Rh B molecules. Additionally, tests using TC instead of Rh B confirmed that the phenomenon was primarily caused by the change in the form of the Rh B molecules rather than by the variation in the Na^+^ ion content of the PTNT materials. This study suggests that the issue of poor adsorption due to pH changes in the Rh B solution can be addressed by reducing the amount of Na^+^ ions in the PTNT materials or by adjusting the pH by adding acidic solutions (such as HCl) to the Rh B solution, within a range that does not affect its properties.

## Figures and Tables

**Table 1:** The 2theta and *d*_200_ values in the XRD pattern and chemical composition of the each PTNT samples calculated based on XRF analysis results.

Sample	2thata (Degree)	*d*_200_ (Å)	Chemical Composition
PTNT-w1	9.3	9.5	Na_0.5_H_1.5_Ti_2_O_5_
PTNT-w2	9.6	9.2	Na_0.2_H_1.8_Ti_2_O_5_

**Table 2:** The specific surface area of PTNT-w1 and PTNT-w2 samples.

Sample	Specific Surface Area (m^2^/g)
PTNT-w1	140.90
PTNT-w2	130.01

**Table 3:** The specific surface area and chemical composition of PTNT-w3 sample.

Sample	Specific Surface Area (m^2^/g)	Chemical Composition
PTNT-w3	145.49	Na_0.1_H_1.9_Ti_2_O_5_

**Table 4:** The rate constant of Rh B photodegradation with PTNT-w1, PTNT-w2, PTNT-w3, and PTNT-w2 + HCl conditions and R-squared value of each kinetic graph in Figure 11.

Sample	Irradiation Light	*k* (s^−1^)	R-Squared Value
PTNT-w1	UV	6.47 × 10^−5^	0.99
	Vis	1.13 × 10^−5^	0.96
PTNT-w2	UV	9.80 × 10^−5^	0.98
	Vis	3.54 × 10^−5^	0.98
PTNT-w3	UV	1.06 × 10^−5^	0.95
	Vis	2.04 × 10^−5^	0.99
PTNT-w2 + HCl	UV	2.19 × 10^−4^	0.99
	Vis	3.20 × 10^−4^	0.99

## Data Availability

The datasets analyzed or generated during this study are available from the corresponding author upon reasonable request.
